# Encoding of the Intent to Drink Alcohol by the Prefrontal Cortex Is Blunted in Rats with a Family History of Excessive Drinking

**DOI:** 10.1523/ENEURO.0489-18.2019

**Published:** 2019-08-23

**Authors:** David N. Linsenbardt, Nicholas M. Timme, Christopher C. Lapish

**Affiliations:** 1 Addiction Neuroscience, Department of Psychology and Indiana Alcohol Research Center, Indiana University–Purdue University Indianapolis, Indianapolis IN 46202; 2 Indiana University School of Medicine Stark Neuroscience Institute, Indianapolis, IN 46202

**Keywords:** alcohol-associated cues, alcohol-preferring rat, electrophysiology, information theory, neural encoding, prefrontal cortex

## Abstract

The prefrontal cortex (PFC) plays a central role in guiding decision making, and its function is altered by alcohol use and an individual’s innate risk for excessive alcohol drinking. The primary goal of this work was to determine how neural activity in the PFC guides the decision to drink. Towards this goal, the within-session changes in neural activity were measured from medial PFC (mPFC) of rats performing a drinking procedure that allowed them to consume or abstain from alcohol in a self-paced manner. Recordings were obtained from rats that either lacked or expressed an innate risk for excessive alcohol intake, Wistar or alcohol-preferring (P) rats, respectively. Wistar rats exhibited patterns of neural activity consistent with the intention to drink or abstain from drinking, whereas these patterns were blunted or absent in P rats. Collectively, these data indicate that neural activity patterns in mPFC associated with the intention to drink alcohol are influenced by innate risk for excessive alcohol drinking. This observation may indicate a lack of control over the decision to drink by this otherwise well-validated supervisory brain region.

## Significance Statement

Aberrant decision-making is both a risk factor for, and the result of, an Alcohol Use Disorder (AUD). Thus, identifying the neural mechanisms regulating drinking decisions may lead to unique prevention and intervention strategies. The current study demonstrates that the intent to drink alcohol is encoded by the activity of neural populations within the medial prefrontal cortex (mPFC). However, the encoding of this intention signal is greatly reduced in a population of rodents with an extensive family history of high alcohol consumption. These data suggest that identifying strategies to restore the contribution of mPFC in the decision to drink alcohol may provide effective targets to treat an AUD, particularly in those with family history of AUDs.

## Introduction

Aberrant decision making is both a risk factor for, and the result of, an alcohol use disorder (AUD; Verdejo-Garcia et al., 2018). Therefore, understanding the neural systems that underlie decision making, and how altered function of these systems influences decisions about drinking alcohol, is critical to identify novel targets to treat and prevent AUDs. While several neural systems have been implicated in decision making, the medial prefrontal cortex (mPFC) plays a critical role in setting goals (Buschman and Miller, 2014) and forming intentions to achieve them (Haynes et al., 2007; Brass et al., 2013; Fuster and Bressler, 2015). Thus, the inability to refrain from excessive drinking may reflect pathology in neural circuits that guide goal-directed actions such as mPFC (Fuster and Bressler, 2015).

Dysfunction of the mPFC has been repeatedly found in populations of subjects that drink alcohol excessively (Schacht et al., 2013). Exposure to experience- or experimentally paired alcohol cues, increases neuronal activity within the PFC (George et al., 2001; Tapert et al., 2003; Kareken et al., 2010), and the magnitude of this effect is correlated with increases in self-reported alcohol craving (Myrick et al., 2004) and relapse (Grüsser et al., 2004). Additionally, recently abstinent individuals with an AUD exhibit reduced baseline neuronal activity within the mPFC (Catafau et al., 1999). Similar effects are observed in rodents, with exposure to alcohol-associated cues eliciting reinstatement of extinguished alcohol seeking and robust increases in biomarkers of neural activity in PFC (Dayas et al., 2007; Groblewski et al., 2012; Pfarr et al., 2015). More recent reports suggest a critical role for the PFC in alcohol extinction learning (Cannady et al., 2017; Keistler et al., 2017), suggesting that this brain region may be critically involved in “remapping” associations between alcohol-associated stimuli and the motivational properties of alcohol. Thus, preclinical rodent and human data converge to implicate altered function of PFC in AUD.

The PFC has also long been known to be involved in the regulation of executive processes required to guide reward-based decision making (Krawczyk, 2002; Ridderinkhof et al., 2004; Bechara, 2005), and animal studies are beginning to shed light on the computational processes that underlie these decisions (Dalley et al., 2004; Fitoussi et al., 2015). Decisions to initiate (or suppress) reward-directed motor actions are encoded in frontal-parietal circuits (Andersen and Cui, 2009), and, in the PFC, the encoding of these actions is evident before action initiation indicating behavioral intent (Sakagami and Niki, 1994; Sakagami and Tsutsui, 1999; Tanji and Hoshi, 2001; Andersen and Cui, 2009; Momennejad and Haynes, 2013; Boulay et al., 2016). These data motivated our hypothesis that similar neurocomputational processes exist in the PFC that regulate alcohol intake decisions. The implications of identifying and understanding processes that underlie the intention to use alcohol cannot be overstated, because intention signals that arise before alcohol seeking/drinking may be particularly effective targets for interventions aimed at reducing or eliminating alcohol consumption.

The data presented herein are novel in-depth analyses of previously published data (Linsenbardt and Lapish, 2015). In this previous study, we assessed neural firing at longer time-scales (e.g., >1 min), which is better suited to detect pharmacologically driven effects. The goal of the current study was to examine neural activity at shorter time scales (e.g., <1 min), to assess decision-making dynamics. To first determine if the signals reflecting the intention to drink alcohol were present in the PFC, the current study evaluated neural activity across populations of neurons recorded during alcohol drinking in well-trained, high drinking, rats. We were particularly interested in the impact of alcohol-associated cues on drinking intent, and the role of family history of alcohol drinking on these cue-elicited decisions, as these factors have been shown to be critically important in human clinical studies (see above) and were previously unexplored. Thus, we used Indiana alcohol-preferring (P) rats, which are a well-validated preclinical model of familial risk for excessive drinking (i.e., “family-history positive”), and a comparison strain with no family history, Wistar rats. We hypothesized that the intention to drink or abstain would be encoded in populations of neurons in the PFC. Furthermore, since individuals with a positive family history display greater PFC responses to alcohol associated stimuli (Tapert et al., 2003; Kareken et al., 2010), we also hypothesized that P rats would display a more robust intention signal compared to Wistar.

## Materials and Methods

### Animals

P rats have been selectively bred for >75 generations for their high drinking phenotype (Li and McBride, 1995; Bell et al., 2006; McBride et al., 2014), and are conceptually analogous to individuals with generations of family history of excessive drinking (i.e., family history positive). As P rats were originally derived from Wistar rats, we opted to use this population (which is “family-history negative”) to assess possible family history effects.

Male P rats (*N* = 22) were ordered from the Indiana Alcohol Research Center Animal Production Core, and male Wistar rats (*N* = 21) were ordered from Envigo. All animals were shipped via truck to our vivarium, and were single housed and placed on a 12/12 h dark/light reverse cycle. Animals were ≈70 d of age before testing and had *ad libitum* access to food and water. All procedures were approved by the Animal Care and Use Committee and conformed to the Guidelines for the Care and Use of Mammals in Neuroscience and Behavioral Research (National Academic Press, 2003).

### Intermittent alcohol procedure (IAP)

The procedural timeline and methods for these experiments have been recently described in detail (Linsenbardt and Lapish, 2015). All animals first underwent an IAP using previously published procedures (Simms et al., 2008): Rats were given access to two bottles, one containing 20% alcohol (v/v) and the other tap water, for 24 h every other day (Monday/Wednesday/Friday) in the home cage. These procedures were continued for four weeks; animals had 12 total 24-h alcohol/water access sessions.

### Two-way cued access protocol (2CAP)

**Figure 1.**
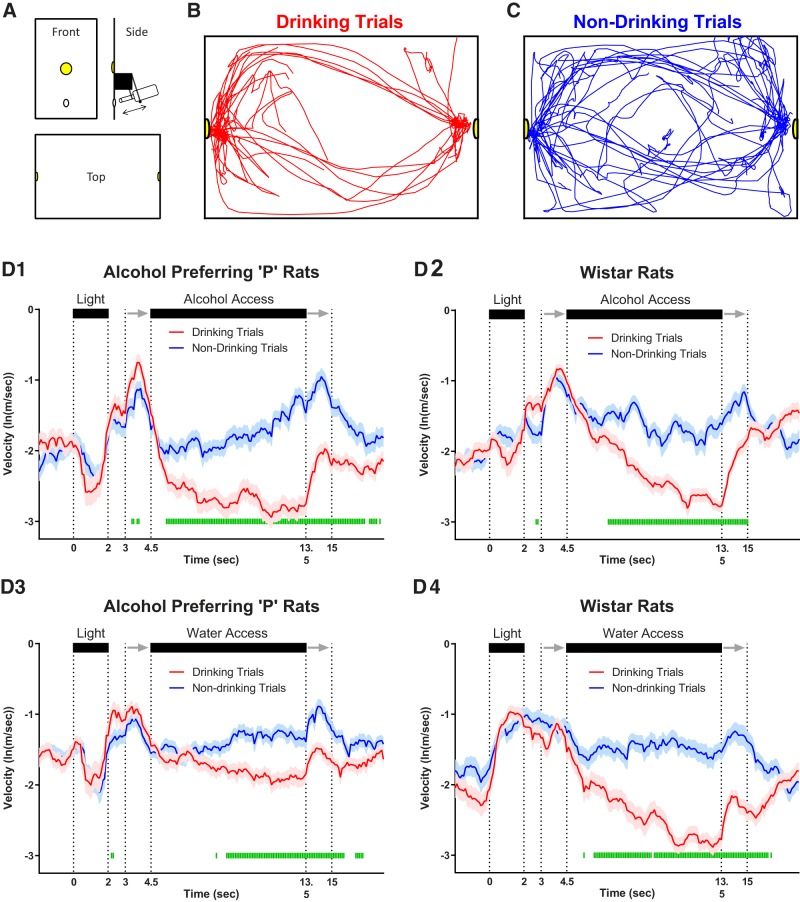
Movement dissociates drinking versus non-drinking trials during fluid availability but not during stimulus (DS; i.e., cue light) presentation. ***A***, Configuration of conditioning boxes used for cue-induced drinking/neurophysiology. Representative traces of head location within the conditioning box on drinking trials (***B***) and non-drinking trials (***C***) from a single session in a Wistar rat given alcohol solution. Illustrations at the top of all figure panels in ***D1–D4*** illustrate the time course of stimuli presentation on each trial. Two seconds of “baseline” data precede the start of each trial, in which a light was illuminated for 2 s on one side of the two-sided chamber. A 1-s “delay” in which no stimuli were activated bridged the light cue and the initiation of sipper movement into the chamber. Sipper movement is represented by the two gray arrows, with the first arrow indicating sipper entry, and the second arrow indicating sipper removal. Fluid was readily available (only on the chamber side cued by the light) between the end of the sipper motor entry (first arrow) and the start of the sipper motor removal (second arrow). ***D1–D4***, Mean (±SEM) log-transformed head movement speed changed significantly over time on drinking trials compared to non-drinking trials in P and Wistar rats on both alcohol and water sessions. Green bars denote drinking versus non-drinking trial differences (FDR-corrected rank-sum tests; *p* < 0.05).

Twenty-four hours following the final (12th) IAP access session animals received access to an unsweetened 10% alcohol (v/v) solution for 2CAP sessions. 2CAP sessions occurred during the dark phase of the light/dark cycle, starting 1–3 h after lights off. The conditioning box configuration is illustrated in Figure 1*A*. During 2CAP, a white stimulus light was illuminated for 2 s on one side of the rectangular box at random. One second after this light was turned off, a sipper tube containing 10% alcohol (v/v) solution was extended into the box on the same side as the light cue. Thus, the light was a Discriminative stimulus (DS+) that predicted the location that the alcohol was to be made available. To ensure the sipper motor sound did not serve as a directional cue, both tube motors were turned on for the same duration, but only the appropriate sipper entered the chamber. The tube was available for ≈10 s. Each trial was separated by a 20- to 180-s intertrial interval (ITI; 90 s on average; randomized order). A total of 40 trials were conducted for 5 out of 7 d/week (weekdays) for three weeks (15 total sessions) before surgery. Water sessions were identical to alcohol sessions except the sippers contained water. During water sessions, a tube containing 10% (v/v) alcohol was present outside the fluid delivery port to ensure that the presence or absence of the alcohol odor did not predict alcohol availability/unavailability.

### Stereotaxic surgery and behavioral electrophysiology

Following the 15-d acquisition/maintenance of 2CAP, a group of Wistar (*N* = 3) and P rats (*N* = 4) with matched 15 d 2CAP alcohol consumption history (P = 1.31 ± 0.06 g/kg; Wistar = 1.25 ± 0.06 g/kg, mean ± SEM) were selected for electrophysiological experiments, and were unilaterally implanted with multi-tetrode arrays in the mPFC (Linsenbardt and Lapish, 2015). This matching was conducted for three principle reasons. First, the use of rats that will reliably consume/self-administer excessive amounts of alcohol under limited access conditions is a prerequisite to identifying how such alcohol consumption alters neurophysiological processes. It is not possible to assess the effects of alcohol consumption in populations that do not drink alcohol. Second, matching for alcohol consumption reduced the possibility that any observed differences in physiology were not simply due to differences in alcohol experience. Finally, these matched populations of rodents are directly comparable to human studies in which groups of family history positive and family history negative individuals are matched for drinking history (Kareken et al., 2010).

After a full recovery from surgery, animals were given a period of one week of habituation/acclimation before electrophysiological recordings. Animals were habituated to the handling required for incremental lowering of tetrodes before the task, and also to navigating the 2CAP environment with the tether connecting the implanted electrode array to the recording hardware. After this habituation period, ≈3 d of 2CAP reinforced with 10% alcohol (v/v) were conducted while electrophysiology was recorded using a 96-channel electrophysiological recording system (Neuralynx). Animals were then given approximately three water sessions where the sippers contained water. A primary goal of these water sessions was to make a direct connection to studies of brain function in humans, wherein alcohol-associated cues are presented in the absence of access to alcohol (Kareken et al., 2010). Electrodes were lowered 50–100 µm before each recording session to collect data from new neuronal ensembles. Following the completion of behavioral testing and electrophysiological recordings, placements were verified via histology (reported in Linsenbardt and Lapish, 2015).

Spike trains were manually identified and sorted into individual cell clusters based on the features of the wave form in Spike Sort 3D (Neuralynx). After cell sorting, duplicate timestamps and interspike intervals <3 ms were removed from spike trains. Only spike trains containing >150 spikes were analyzed.

### Video tracking/behavioral monitoring

One video camera was used in conjunction with ANY-maze software to track the head location of animals while they performed the task, and another was used to record high-definition video and audio to identify trials where animals ultimately consumed fluid (drinking trials; Fig. 1*B*) or did not (non-drinking trials; Fig. 1*C*). Drinking trials were assessed offline and defined as trials where at least one “lick” occurred. A lick was detected by the combination of the animals behavior and the sound of the ball-bearing sippers that were clearly audible in the video recordings. Digital *xy* coordinates were converted to voltage and fed directly into electrophysiology hardware where they were recorded in parallel to neuronal activity. Raw tracking values were used to plot the location of the animals within the conditioning apparatus.

### Experimental design and statistical analysis

#### Behavioral statistics

Detailed behavioral results for animals used in electrophysiology studies were recently described (Linsenbardt and Lapish, 2015). Behavioral analyses for the current work were primarily focused on time-locked changes in locomotor behavior in response to the various task stimuli. We were particularly interested in determining if there were differences in behavior between trials in which animals ultimately drank fluid, or did not, as these differences may be related to (or mediated by) computations in the PFC encoding drinking decisions. Head movement speed was positively skewed, so it was first log transformed to normalize. We next evaluated differences between movement speed on a bin-by-bin basis using rank-sum tests, which were followed by Benjamini–Hochberg false discovery rate (FDR) correction for multiple comparisons. The number of drinking and non-drinking trials were analyzed using two-way repeated measures ANOVA with rat population (P vs Wistar) as the between groups factor and number of drinking/non-drinking trials as the within-subjects factor.

#### General electrophysiology statistics

The results of firing rate over the course of the entire 2CAP sessions for electrophysiology studies were recently described (Linsenbardt and Lapish, 2015). The primary goal herein was to evaluate *cue-induced* alterations in neural activity, which was not evaluated previously. Peristimulus time histograms (PSTHs) were created by aligning binned (100 ms) spike trains for each neuron to the onset of each trial. PSTHs were smoothed using a Gaussian function with a standard deviation of 300 ms, and softmax normalized to avoid being biased by high firing rate neurons by dividing the firing rate of each neuron by its maximum variance (Ames et al., 2014).

#### Stimuli/task responsiveness of individual neurons

A signal-to-noise statistic (d´) was used to quantify the degree to which each neurons activity changed in response to the task stimuli compared to pre-task (baseline) activity as well as chance (surrogate testing); binned (100 ms) spike trains were not transformed or normalized in any way before these analyses. Individual neurons were evaluated for the degree of responsiveness using d´ (Barr et al., 2010; Gale and Perkel, 2010). Specifically, d´ was calculated by dividing the absolute values of the mean difference between firing rate during the baseline epoch and the rest of trial by the square root of the sum of their squared deviations. To evaluate the significance of the d´ values, surrogate data were created by taking each neurons spike train and randomly shuffling it 500 times; d´ was then determined for each of the 500 randomly shuffled spike trains and these values were used to compute a 95% confidence interval of the null distribution for each neuron. To evaluate differences in d´ on drinking versus non-drinking trials, a two-way ANOVA was conducted with responsiveness group (drinking significant, non-drinking significant, both significant) and trial type (drinking and non-drinking trials) as factors, which was followed by Sidak-corrected *post hoc* comparisons. To evaluate proportions of responsive neurons in P versus Wistar rats, χ^2^ analyses were conducted on alcohol and water sessions separately.

#### Mutual information (MI) of individual neurons

Following d´ analyses, we next used MI (an information theoretic statistical approach; Cover and Thomas, 2005; Timme and Lapish, 2018) to precisely quantify the total amount of information encoded by each neuron. This approach is preferable to other parametric statistical analysis of firing rate, as firing rate distributions are highly non-normal (Roxin et al., 2011; Timme et al., 2016). We focused these analyses on two categorical domains, the amount of information encoding real trials versus null trials (collectively referred to as trial-encoding), and the amount of information encoding drinking trials versus non-drinking trials (collectively referred to as drink-encoding). Null trials were constructed from periods of the neural recording that were randomly selected from the ITI such that full null trials did not overlap real trials at any time.

We began the MI calculation by aligning the first 10 drinking, the first 10 non-drinking, and 20 null trials relative to the cue onset (drinking and non-drinking trials) or a randomly chosen time point during the ITI (null trials). Null trials were constructed from periods of the neural recording that were randomly selected from the ITI such that full null trials did not overlap real trials at any time. At a given time bin *t* relative to the stimulus onset time and for a given neuron *i*, we constructed a joint discrete probability distribution p_i,t_(*x*,*y*), where *x* was the discretized smoothed spiking rate of the neuron (see above, 100-ms bins relative to stimulus onset) and *y* was either the trial type (real vs null) or the drink outcome (drinking trials vs non-drinking trials). The smoothed spiking rate of the neuron was discretized such that the values across trials were ranked and binned into three states (low, medium, or high firing) with equal number of counts (or as close to equal as possible in the event of tied values). The probability was then calculated by dividing the number of joint state observations by the total number of trials. For instance, in the case of drink outcome encoding, for a given neuron and time bin, we may have observed five joint states in which the neuron had a low firing rate during drinking trials. In this example, p_i,t_(*x* = low,*y* = drinking) = 5/20 = 0.25. In the case of stimulus encoding, we might have observed seven joint states in which the neuron had a high firing rate during real trials. In this example, p_i,t_(*x* = high,*y* = real) = 7/40 = 0.175. Note that drink encoding only used real trials, so only 20 total observations where performed, whereas trial encoding used both real and null trials, resulting in 40 total observations.

For each neuron, time bin, and encoding type (drink encoding and trial encoding), we calculated the MI using Equation 1:

(1) $$I_{i,t}\left(x,y\right)=\underset{x,y}{\sum }p_{i,t}\left(x,y\right)log_{2}(\frac{p_{i,t}\left(x,y\right)}{p_{i,t}\left(x\right)p_{i,t}\left(y\right)}).$$

We used base 2 for the logarithm in Equation 1 to produce MI results in units of bits. In Equation 1, the marginal discrete distributions p_i,t_(x) and p_i,t_(y) are found by summing over the other variable:

(2) $$p_{i,t}\left(x\right)=\underset{y}{\sum }p_{i,t}\left(x,y\right),\;p_{i,t}\left(y\right)=\underset{x}{\sum }p_{i,t}(x,y).$$

The MI quantifies how much information one variable provides about the other. In this case, if a neuron tends to fire much more frequently on drinking trials than non-drinking trials, for instance, a large MI value would result. However, if drinking status and neuron firing rate were unrelated, then a small MI value would result. By calculating the MI at each time bin for each neuron, we were able to evaluate encoding dynamically throughout the task.

Due to the discrete nature of experimental trials and the fact that MI results cannot be lower than 0, noise tends to bias MI results upwards (Treves and Panzeri, 1995; Panzeri et al., 2007). To assess the likelihood that a given MI result is not simply the result of noise, we calculated a *p* value for each MI result by randomizing the joint observations 100 times and recalculating the MI for these null surrogates. The randomization procedure preserved the marginal distributions. The *p* value was then calculated as the proportion of null surrogates with a MI result greater than or equal to the observed value in the real data. In the case where all null MI values were less than the result from the real data, the *p* value was set to 0.005 = 0.5 × (1/100) due to the resolution associated with using 100 null surrogates.

Next, to ensure that non-significant MI values did not inflate the estimates of SE, the *p* values were used to calculate a weight (w) for each MI result via w = -log_10_(p). These weights were then normalized by dividing each weight by the sum of all the weights and used to calculate the weighted mean (Eq. 3) and SE of the weighted mean (Eq. 4) across all relevant neurons (animal strain and liquid type) at a given time bin t.

(3) $${\overset{¯}{I}}_{t}\left(x,y\right)=\underset{i}{\sum }w_{i}I_{i,t}(x,y).$$

(4) $$SEM_{t}\left(x,y\right)=\sigma_{t}\sqrt{\underset{i}{\sum }w_{i}{}^{2}}.$$

In Equation 4, σ_t_ is the standard deviation of the MI values across all neurons at the given time bin t. Therefore, large MI values that were unlikely to be due to chance received large weights and factored heavily into the weighted mean. In the case where the MI results had similar weights, the SE of the weighted mean approached the SEM. In the case where the MI results were dominated by a few highly weighted values, the SE of the weighted mean approached the standard deviation.

While the weighting procedure above allowed us to highlight the importance of significant information results, in time bins where few significant information results were observed, an upwards bias in the information results would still be observed. To detect cases where the ensemble of information values was not significantly different from null, we also used a Kolmogorov–Smirnov test to compare the distribution of real MI results to the distribution of MI results from null surrogate data used to calculate the individual neuron *p* values. This allowed us to assess the time bins for which the entire ensemble of neurons was not significantly different from null data, suggesting the ensemble as a whole was not encoding significant amounts of information (open circles in figures). We applied a threshold of *p* < 0.01 to all such Kolmogorov–Smirnov tests to assess significant ensemble encoding.

Finally, to compare information results between animal populations (P vs Wistar), we used a bootstrap approach to compare the weighted mean MI between P and Wistar rats at each time bin. We compared the difference between the weighted mean MI values in the real data to the difference weighted mean MI results from 10,000 randomized trials (identity of P and Wistar neurons randomized preserving number of neurons in each group). The *p* value was then calculated as the proportion of randomized trials with differences greater than or equal to the difference in the real data, accounting for the sign of the difference. In the case where all randomized trial difference values were less than the result from the real data, the *p* value was set to 0.00005 = 0.5 × (1/10,000) due to the resolution associated with using 10,000 randomized trials. These *p* values were then corrected for multiple comparisons across time bins within a given figure using FDR control (Benjamini and Hochberg, 1995).

#### Principle component analysis (PCA)

PCA was conducted to evaluate the predominant population-level firing rate dynamics. PCA is commonly used as a dimensionality reduction tool that requires minimal assumptions of the data (Cunningham and Yu, 2014). A single “omnibus” PCA was conducted on a matrix containing all data for all groups so that every possible comparison could be made statistically. This matrix included ensemble activity on drinking trials, non-drinking trials, and equally sized, randomly sampled data vectors (previously described null trials).

#### Neural population state-space (SS) analyses

For SS analyses, neural population activity was projected onto the first three PCs of PCA space. These analyses allowed us to determine the time course of alterations in the pattern of firing rate. Similar patterns of population activity reside close to each other in 3-dimensional space, and when different are further apart. Differences in distance between 3-dimensional population activity vectors were evaluated on a bin-by-bin basis via Euclidean distance analyses (Ames et al., 2014). The mean distance between each trial and every other trial in that comparison type were made (for example drinking trial 1 vs all null trials, drinking trial 2 vs all null trials, etc.), and the mean and variance of the (non-redundant) distances were used for plotting and statistical analyses. We were specifically interested in differences between drinking and non-drinking trials (vs null trials), and therefore evaluated Euclidean distance between these groups and null trials on a bin by bin bases using Benjamini–Hochberg FDR-corrected rank-sum testing.

## Results

### Movement dissociates drinking versus non-drinking trials during fluid availability but not during stimulus (DS) presentation

A summary of statistics for each figure is provided in table format (Table 1). To assess the neural dynamics of alcohol-associated cues within mPFC, extracellular electrophysiological activity was obtained from ensembles of neurons during performance of an alcohol-drinking task in Wistar and P rats matched for alcohol history (McCane et al., 2014; Linsenbardt and Lapish, 2015). Neural recordings were performed in well-trained animals that had more than seven weeks of prior alcohol experience. Subsequent recordings were made using identical procedures, except the alcohol solution was replaced with water. The layout of the conditioning apparatus (Fig. 1*A*), as well as representative video tracking data on drinking (Fig. 1*B*) and non-drinking (Fig. 1*C*) trials are presented in Figure 1. Head movement speed differentiated drinking from non-drinking trials in both rat populations on both alcohol and water sessions, primarily (or exclusively) during the fluid access epoch (FDR-corrected rank-sum tests; *p* < 0.05; Fig. 1*D*). Differences during fluid access were expected, as drinking required that animals remain in close proximity to the sipper on drinking trials. No differences in movement speed were observed during the DS of drinking versus non-drinking trials, while transient differences were observed from the 2- to 4.5-s period following DS offset (Fig. 1*D*). Furthermore, no differences (main effects or interactions; *p* > 0.20) were observed in the mean number of drinking (number of trials ± SEM: P = 19.19 ± 1.31; Wistar = 22.40 ± 2.03) or non-drinking trials (number of trials ± SEM: P = 20.50 ± 1.34; Wistar = 17.60 ± 2.03). Collectively these data indicate that the behavioral response to the DS was not predictive of a drinking trial.

### Task stimuli elicited differential responsiveness in neurons on drinking trials versus non-drinking trials

**Figure 2.**
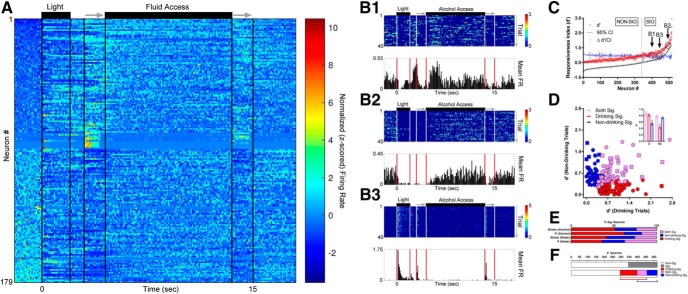
Task stimuli elicited varied responses in neurons on drinking trials versus non-drinking trials, illustrating the capacity to encode/predict future drinking. ***A***, The z-scored time course of alterations in firing rate in each of the 179 neurons with significant firing rate alterations (ignoring drinking vs non-drinking status) sorted from lowest baseline firing rate (top) to highest baseline firing rate (bottom). ***B1–B3***, PSTHs of three representative neurons recorded from a Wistar rat during the same alcohol access session; all displayed significant alterations in firing rate (see panel ***C***). ***C***, Approximately 1/3 of all neurons displayed significant alterations in firing rate versus baseline as measured by d´ (ignoring drinking versus non-drinking status). ***D***, Significant individual neuron d´ scores on only drinking trials (red), only non-drinking trials (blue), and both drinking and non-drinking trials (purple). Square symbols represent data from Wistar rats and circle symbols represent data from P rats. The mean of d´ scores on drinking versus non-drinking trials from these subgroups was as expected (inset); drinking-responsive neurons had lower d´ values on non-drinking trials, and non-drinking-responsive neurons had lower d´ values on drinking trials (two-way ANOVA; *F*_(2,679)_ = 38.03, *p* < 0.0001; asterisks in inset indicate significantly lower d´ scores from other two comparison groups (Sidak’s multiple comparisons adjusted *p* < 0.01). ***E***, The proportion of neurons displaying significant d´ values (drinking/non-drinking/both) were similar between P and Wistar rats (χ^2^ *p* ≥ 0.20). ***F***, When data were evaluated independently of drinking status (top), a smaller proportion of neurons demonstrated selectivity to presentation of environmental stimuli (≈33%) than when selectivity was assessed taking drinking/non-drinking trials into account (bottom, ≈43%).

To determine if firing rates of individual neurons differed on drinking versus non-drinking trials, the changes in firing evoked by the presentation of trial-associated stimuli (e.g., DS, sipper) was compared to a baseline period 2 s immediately before the trial (Fig. 2*A*). Heterogeneity in the firing rates evoked by task stimuli varied greatly between neurons, with some showing both increases and decreases in firing rate (Fig. 2*B1*), and others displaying only decreases (Fig. 2*B2*) or increases (Fig. 2*B3*). The signal-to-noise statistic d´ was used to identify stimulus responsive neurons, and out of 520 neurons across both groups of rats, 179 (≈34%) displayed statistically significant changes in d´ (Fig. 2*A*,*C*). Neurons were observed that responded to task-associated stimuli similarly on drinking and non-drinking trials (Fig. 2*D*, purple group). Additionally, subgroups of neurons were then identified that were influenced by task stimuli only on drinking trials or non-drinking trials (Fig. 2*D*, red and blue groups, respectively). Comparisons of drink-encoding neurons confirmed that non-drinking trial responsive neurons displayed lower responsiveness on drinking trials. The converse was also true; the subgroup of drinking trial responsive neurons displayed lower responsiveness on non-drinking trials (two-way ANOVA; *F*_(2,679)_ = 38.03, *p* < 0.0001; Fig. 2*D*, inset). Interestingly, a greater number of responsive neurons were found when drinking status was taken into account compared to when it was ignored (225 vs 179; Fig. 2*F*), with no significant differences in the proportions of neuron response between P and Wistar rats on either alcohol (χ^2^ = 3.24; *p* = 0.20) or water sessions (χ^2^ = 2.34; *p* = 0.31; Fig. 2*E*). Thus, mPFC neurons were found that possessed the capacity to encode decisions and/or behaviors associated with drinking/non-drinking trials.

### P rats exhibit diminished drink encoding

To quantify and compare the *amount* of information encoded by trial- and drink-encoding neurons over time, information theoretic statistical approaches were used. The goal of these analyses were to capture the amount of information encoded in each neuron about the trial-associated stimuli (trial-encoding) and if the neural firing rates dissociated drinking/non-drinking trials (drink-encoding). Additionally, these analyses focused on drink-encoding that occurred before fluid availability (the 0- to 4.5-s interval), as this time interval was expected to contain cue-elicited encoding of the intention to drink or abstain. In addition to quantifying the amount of information using MI, these analyses captured different encoding strategies (e.g., firing rate increases or decreases) at each time bin during a trial (e.g., encoding during the DS vs encoding during access).

**Figure 3.**
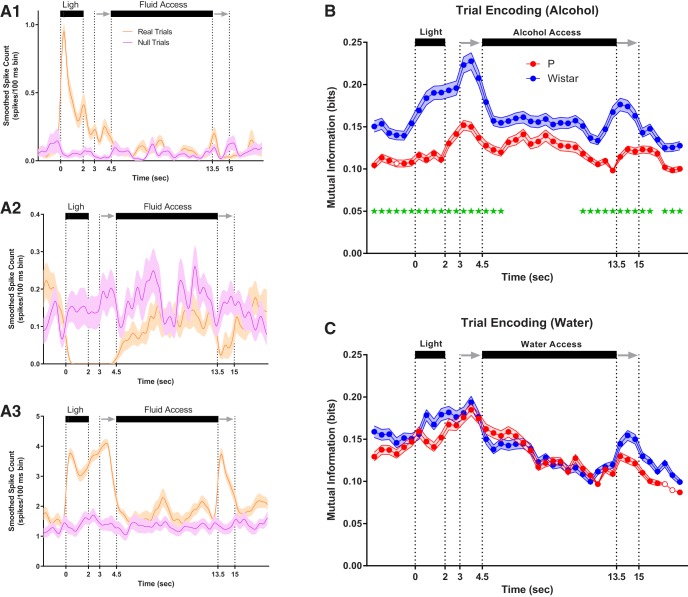
P rats exhibit blunted trial encoding during alcohol sessions. ***A1–A3***, Mean firing rate of three representative trial encoding neurons. Neurons in ***A1***, ***A3*** (Wistar/alcohol and P/water) encoded trial stimuli with increases in firing rate, whereas neuron in ***A2*** (Wistar/alcohol) did so with decreases in firing rate. Neurons displayed significant heterogeneity in the magnitude and location of trial encoding. For example, neurons in ***A1***, ***A3*** displayed differences in the encoding of the sipper retracting. Also, ***A2***, ***A3*** encode both visual and auditory stimuli. On average, Wistar neurons encoded more information about trial stimuli than P during alcohol sessions (***B***), whereas no differences were observed between P and Wistar during water sessions (***C***). Data represent weighted mean ± SE of the weighted mean. Green asterisks represent FDR-corrected differences between P and Wistar (*p* < 0.01). Open circles represent time bins where the ensemble of neurons did not produce significant encoding.

Examples of trial-encoding neurons are plotted in Figure 3*A1–A3*. There was marked heterogeneity in trial-encoding. The neurons in Figure 3*A1*,*A3* encoded trial stimuli with increases in firing rate, whereas the neuron in Figure 3*A2* did so with decreases in firing rate. The neurons in Figure 3*A1*,*A3* displayed differences from one another in the encoding of the sipper retracting. Additionally, the neurons in Figure 3*A2*,*A3* encoded both visual (light) and auditory stimuli (sipper entry), compared to the neurons in Figure 3*A1*, which primarily encoded visual (DS) stimuli. Collectively, the neurons recorded from Wistar rats exhibited stronger trial-encoding than P rats during alcohol sessions (FDR-corrected rank-sum tests; *p* < 0.05; Fig. 3*B*), whereas no differences were observed in trial-encoding during water sessions (FDR-corrected rank-sum tests; *p* < 0.05; Fig. 3*C*).

**Figure 4.**
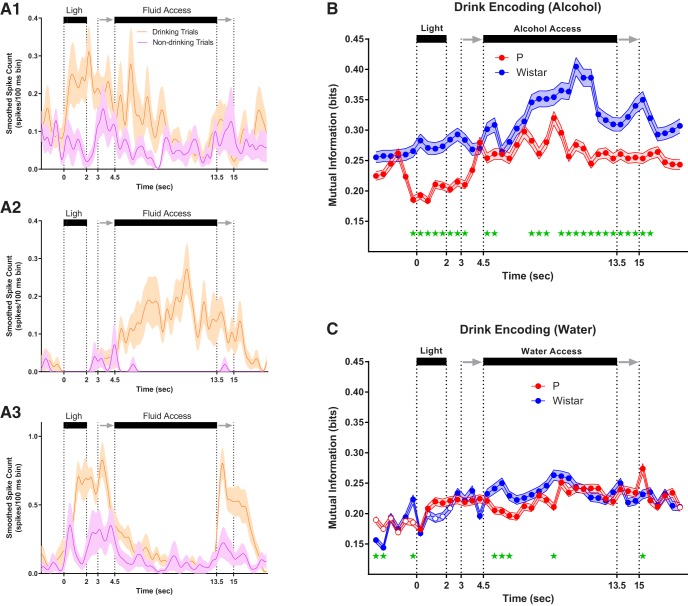
P rats exhibit diminished drink encoding during alcohol sessions. ***A1–A3***, Mean firing rate of three representative drink encoding neurons. Neurons in ***A1***, ***A3*** (Wistar/water and Wistar/alcohol) encoded drinking intent (pre-fluid availability drink encoding), whereas neuron in ***A2*** (P/alcohol) encodes drinking only during fluid availability. As with trial encoding, neurons displayed significant heterogeneity in the magnitude and location of drink encoding. For example, neurons in ***A1–A3*** displayed differences in the encoding of drinking during/following fluid removal. On average, Wistar neurons encoded more information about drinking/non-drinking than P during alcohol sessions (***B***), whereas inconsistent/transient differences were observed between P and Wistar during water sessions (***C***). Data represent weighted mean ± SE of the weighted mean. Green asterisks represent FDR-corrected differences between P and Wistar (*p* < 0.01). Open circles represent time bins where the ensemble of neurons did not produce significant encoding.

Examples of drink-encoding neurons are plotted in Figure 4*A1–A3*. As with trial-encoding, neurons displayed heterogeneity in the magnitude and location of drink-encoding. The neurons in Figure 3*A1*,*A3* encoded drinking intent (pre-fluid availability drink-encoding), whereas the neuron in Figure 4*A2* encoded drinking only following fluid availability. The neurons in Figure 4*A1–A3* displayed differences from one another in the encoding of drinking during/following fluid removal. Collectively, neurons recorded from Wistar rats encoded more information than P rats about drinking/non-drinking trials before alcohol access versus P rats (FDR-corrected rank-sum tests; *p* < 0.05; Fig. 4*B*), which may indicate that the mPFC of Wistar rats performed computations associated with subsequent drinking; such as the intention to drink. In contrast, there were little to no differences in drink-encoding across rat populations before water availability (FDR-corrected rank-sum tests; *p* < 0.05; Fig. 4*C*).

### Neural activity patterns in populations of mPFC neurons reflect the intention to drink in Wistar, but not P, rats

**Figure 5.**
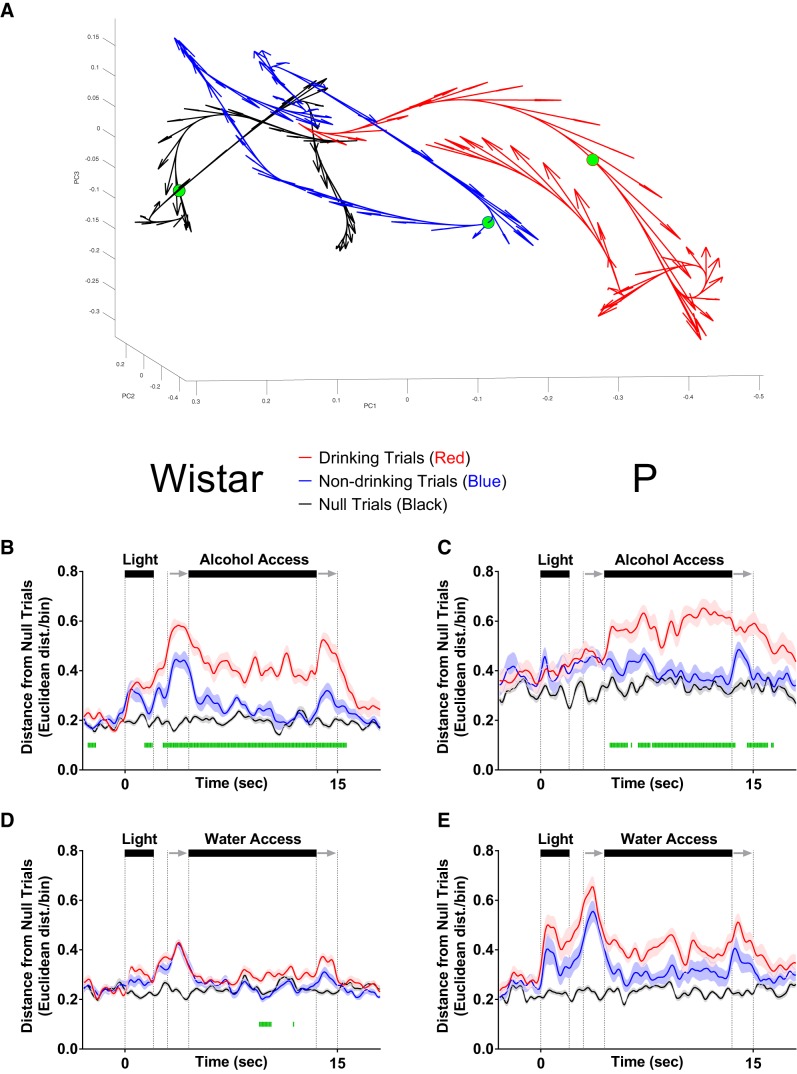
mPFC neural activity patterns reflect the intention to drink alcohol in Wistar, but not P, rats. ***A***, Illustrates neural trajectories in 3-dimensional Euclidean space on a single drinking (red), non-drinking (blue), and null trial (black). Filled green circles indicate the same time bin across each of the conditions, with the Euclidean distance between drinking (0.67) and non-drinking (0.59) trials from null used for statistical analyses in ***B–E***. ***B***, Populations of neurons in Wistar rats on alcohol access sessions encoded the intent to drink or not drink; differences in the pattern of firing between drinking/non-drinking trials were observed before alcohol access. ***C***, Populations of neurons in P rats on alcohol access sessions encoded drinking/non-drinking but did not encode alcohol drinking intent. ***D***, Populations of neurons in Wistar only transiently encoded water drinking. ***E***, Populations of neurons in P failed to encode water drinking or water drinking intent. Data are presented as mean ± SEM. Green lines represent FDR-corrected differences in Euclidean distance between drinking and non-drinking trials (*p* < 0.05).

**Figure 6.**
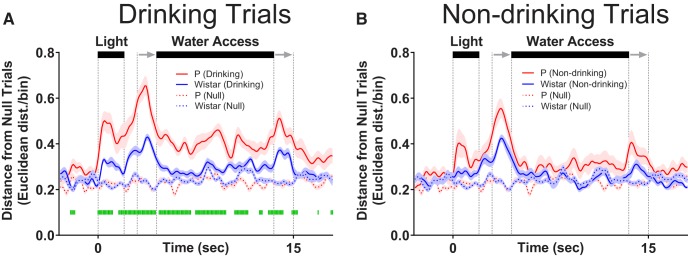
mPFC neural activity patterns more robustly encode alcohol-associated stimuli than Wistar during water sessions. Data presented in this figure are identical to those found in Figure 5*D*,*E* and are presented here to illustrate P versus Wistar differences. ***A***, On drinking trials during water sessions, population of neurons in P rats better encoded alcohol-associated task/stimuli than Wistar rats, whereas there were no differences in encoding of task/stimuli between P and Wistar on non-drinking (water) trials (***B***). Data are presented as mean ±SEM. Green lines represent FDR-corrected differences between P and Wistar (*p* < 0.05).

To determine whether differences in information encoding observed at the single neuron level were maintained at the population level, SS analyses were performed to quantify how neural activity patterns, captured via principle components, evolved throughout a trial. To quantify the evolution of neural trajectories, Euclidean distance to a corresponding time bin of a null trial was computed for drinking, non-drinking, and null trials (note: a given null trial was compared to all other null trials to compute distance). Euclidean distance was calculated from a multidimensional space that was defined by the first 3 principle components. Larger values of Euclidean distance correspond to larger differences in neural activity patterns, which indicate that the predominant patterns of neural firing were different for two comparisons (Fig. 5*A*). Videos 1, 2, 3, 4 for each comparison group are provided to illustrate the evolution of neural trajectories over time for each trial. During alcohol sessions, alcohol-associated cues elicited neural activity patterns that diverged before the availability of alcohol when drinking versus non-drinking trials were compared in Wistar (FDR-corrected rank-sum tests; *p* < 0.05; Fig. 5*B*), but not P rats (FDR-corrected rank-sum tests; *p* < 0.05; Fig. 5*C*). In other words, the temporal evolution of neural activity patterns in Wistar rats in response to alcohol-associated cues were predictive of future drinking/non-drinking trials, whereas the neural activity patterns in P rats were not. Additionally, during water sessions, population activity only briefly differentiated drinking trials from non-drinking trials in Wistar (FDR-corrected rank-sum tests; *p* < 0.05; Fig. 5*D*), and failed entirely in P rats (FDR-corrected rank-sum tests; *p* < 0.05; Fig. 5*E*). In contrast, there were large differences between P and Wistar in cue/task-elicited population activity. Specifically, on water drinking trials, P rats displayed greater alterations in neural activity patterns versus Wistar rats (FDR-corrected rank-sum tests; *p* < 0.05; Fig. 6*A*). Thus, in P rats, the mPFC was biased toward encoding alcohol drinking during alcohol consumption, whereas in Wistar rats, encoding of the intention to drink alcohol and alcohol drinking was present. Therefore, converging evidence suggests that the encoding of alcohol drinking intent is impaired in the mPFC of P rats, which may contribute to the predisposition for excessive alcohol consumption.

Video 1. Data from Wistar rats given alcohol access. The top panel in all videos is identical to data found in Figure 6. The bottom panel was generated using DataHigh software (Cowley et al., 2013) and represents the time course of neural trajectories over the course of drinking trials (red), non-drinking trials (blue), and null trials (black), in 3-dimensional (Euclidean) space. 10.1523/ENEURO.0489-18.2019.video.1

Video 2. Data from P rats given alcohol access. The top panel in all videos is identical to data found in Figure 6. The bottom panel was generated using DataHigh software (Cowley et al., 2013) and represents the time course of neural trajectories over the course of drinking trials (red), non-drinking trials (blue), and null trials (black), in 3-dimensional (Euclidean) space. 10.1523/ENEURO.0489-18.2019.video.2

Video 3. Data from Wistar rats given access to water. The top panel in all videos is identical to data found in Figure 6. The bottom panel was generated using DataHigh software (Cowley et al., 2013) and represents the time course of neural trajectories over the course of drinking trials (red), non-drinking trials (blue), and null trials (black), in 3-dimensional (Euclidean) space. 10.1523/ENEURO.0489-18.2019.video.3

Video 4. Data from P rats given access to water. The top panel in all videos is identical to data found in Figure 6. The bottom panel was generated using DataHigh software (Cowley et al., 2013) and represents the time course of neural trajectories over the course of drinking trials (red), non-drinking trials (blue), and null trials (black), in 3-dimensional (Euclidean) space. 10.1523/ENEURO.0489-18.2019.video.4

## Discussion

The goal of the current study was to determine if the intent to drink alcohol was encoded by populations of neurons in the rodent mPFC, and if such encoding was influenced by a family history of alcohol drinking. Task-stimuli-evoked changes in neural activity were observed in mPFC of both strains of rats (Fig. 2). Contrary to our hypothesis, during alcohol sessions, patterns of neural activity at both the single neuron and population levels more robustly disambiguated drinking from non-drinking trials in Wistar rats. Importantly, these differences were observed before the availability of alcohol, possibly reflecting the intent to drink (Figs. 4*B*, 5*B*). Additionally, during alcohol sessions, enhanced trial-encoding was observed in Wistar rats (Fig. 3*B*), whereas during water sessions, task-stimuli-evoked changes in neural population activity was larger in P rats (Fig. 6*A*). Collectively, these data suggest that differences in family history of excessive drinking may alter the computations performed by mPFC that control alcohol drinking, either directly, or as a consequence of an interaction between inherited/genetic differences and moderate (but similar) alcohol history.

### In water sessions, P rats more robustly encode alcohol-associated stimuli

P rats are an extremely well-validated rodent model of AUD (Waller et al., 1982; Lumeng and Li, 1986; Gatto et al., 1987; Stewart et al., 1991; McBride and Li, 1998; Bell et al., 2014; McBride et al., 2014; Kampov-Polevoy et al., 2000). One feature that sets these animals apart from other rodent populations that willingly consume alcohol, is their robust seeking phenotype (Czachowski and Samson, 2002). Given this, it was surprising to find that during alcohol seeking/consumption, trial-encoding was weaker in P rats (Fig. 3*B*). Weaker trial-encoding in P rats did not result in an opportunity cost, as there were no differences in the number of drinking trials or volume of fluid consumed between Wistar and P rats. However, differences in drinking were intentionally minimized across Wistars and P rats, as each animal was selected to control for differences in behavior and, especially, history of alcohol intake (Linsenbardt and Lapish, 2015). Since differences in trial-encoding were not associated with increased seeking or drinking when reinforced with alcohol, it does not likely reflect the motivational salience of the stimuli or more basic features of the stimuli such as its perceived intensity or information required to locate/time the delivery of the reinforcer.

Several studies have found that P rats display persistent alcohol-seeking behavior in the presence of cues previously associated with alcohol access/consumption compared to other strains (Ciccocioppo et al., 2001; Czachowski and Samson, 2002; Linsenbardt and Lapish, 2015). This suggests that alcohol-associated stimuli retain their motivational properties in P rats when alcohol is not available. Consistent with this hypothesis, the only comparison where P rats exhibited stronger encoding than Wistars to cues preceding fluid availability was during water sessions at the neural population level (Fig. 6*A*). This observation may reflect the conflict-driven recruitment of mPFC in response to the violation of the previously acquired association between trial-associated stimuli and alcohol experience. Alternatively, enhanced stimuli-encoding during water sessions may reflect the “cached” value of alcohol-associated cues based on prior experiences with alcohol/stimuli rather than their current value (Daw et al., 2005; Doya, 1999; Rangel et al., 2008; Redish et al., 2008; Dezfouli and Balleine, 2013). Consistent with this, an enhanced BOLD response to alcohol associated stimuli in those with increased familial risk for an AUD versus those not at risk has only been observed in a similar setting in which alcohol-associated stimuli are presented in the absence of alcohol access/exposure (Kareken et al., 2010).

### Encoding of the intent to drink alcohol in mPFC is diminished in P rats

In the current study, the encoding of drinking intent (e.g., drink outcome specific changes in neural activity before drinking) was diminished in mPFC of P rats compared to Wistar rats. While, these data are the first to provide evidence that mPFC neurons directly encode the intent to consume alcohol, they also indicate this signal is diminished in animals with increased risk of excessive drinking. These data suggest that increased familial risk diminishes the contribution of the mPFC in the decision to seek and drink alcohol. However, there is also substantial evidence for transitions in encoding in subcortical brain regions, such as the striatum. The dorsomedial striatum directly influences alcohol consumption that is still sensitive to devaluation (i.e., not “habitual”), whereas the dorsolateral striatum modulates alcohol consumption only after prolonged training in which animals have become insensitive to devaluation and display habitual behavioral responding (Corbit et al., 2012). Thus, over the course of repeated alcohol drinking experiences, there is a reorganization of the neural circuits that regulate alcohol drinking behavior. Taken together, these studies underscore the need to disambiguate the distinct roles played by alcohol and family history on the neural circuits that regulate devaluation insensitive and/or aversion-resistant drinking; issues not directly addressed in the current studies.

### Summary/conclusions

Collectively, the data provided herein indicate differences in the role of the mPFC in alcohol consumption between populations with or without increased familial risk of excessive drinking. This finding is characterized by two primary features observed in P rats. (1) The encoding of the decision to drink is blunted during alcohol drinking. (2) And the encoding of stimuli previously associated with alcohol is enhanced during water sessions. We have also observed that neurons in the mPFC of P rats may be uniquely sensitive to the alcohol-associated context of the 2CAP conditioning chamber (i.e., before task; Linsenbardt and Lapish, 2015). The expression of these features was observed in animals that exhibit an inherited risk for excessive drinking, which may reflect underlying differences in neurobiology that facilitate persistent alcohol seeking and/or the transition to aversion-resistant drinking. Identifying strategies to restore the contribution of the mPFC in the intention to drink alcohol and blunt the encoding of alcohol associated cues observed in water sessions may provide effective targets to treat an AUD. Importantly these data further highlight the need to consider inherited/genetic risk factors when developing treatment strategies for AUDs.

**Table 1.** Detailed statistics summary

	Figure	Comparison	Data structure	Type of test	Statistic	Confidence, 95% CI
a	1*D1–D4*	Drinking vs non-drinking movement	Non-normal	FDR-corrected rank-sum		*p* < 0.05
b	2*C*	Neuron responsiveness	Normal	d´		*p* < 0.05
c	2*D*, inset	Neuron responsiveness (drinking vs non-drinking)	Normal	Two-way ANOVA	Df = 2; *F* = 38.03	*p* < 0.0001
d	2*E*	Neuron responsiveness proportions: alcohol	Normal	χ^2^	χ^2^ = 3.24	*p* = 0.20
e	2*E*	Neuron responsiveness proportions: water	Normal	χ^2^	χ^2^ = 2.34	*p* = 0.31
f	3*B*,*C*	Trial encoding	Non-normal	FDR-corrected rank-sum		*p* < 0.05
g	4*B*,*C*	Drink encoding	Non-normal	FDR-corrected rank-sum		*p* < 0.05
h	5*B–E*	Neural population SS	Non-normal	FDR-corrected rank-sum		*p* < 0.05
I	6	Neural population SS	Non-normal	FDR-corrected rank-sum		*p* < 0.05

## Synthesis

Reviewing Editor: Karen Szumlinski, University of California at Santa Barbara Decisions are customarily a result of the Reviewing Editor and the peer reviewers coming together and discussing their recommendations until a consensus is reached. When revisions are invited, a fact-based synthesis statement explaining their decision and outlining what is needed to prepare a revision will be listed below. The following reviewer(s) agreed to reveal their identity: NONE.

Following consultation with 2 reviewers with expertise in the functional neuroanatomy and neurophysiology of alcoholism-related behavior, we have reached the decision of Revise and Re-review on your manuscript. Both reviewers and I agreed that the data summarized in this well-written report are novel and we all appreciated the sophistication of the experimental approaches, including the use of information theory to analyze the data. To the best of our knowledge, this study is the first to record from neurons in the PFC to examine their firing pattern in relation to alcohol cue-elicited approach behavior and the comparison of PFC activity between P and Wistar rats provides novel insight into a potential relation between genetic vulnerability to alcoholism, the appetitive motivational value of alcohol-associated cues, and disrupted PFC function. However, both reviewers raised a relatively large number of what I consider to be valid concerns regarding the report that we would like to see addressed prior to publication. None of the concerns require further experimentation and thus, we feel that the revisions could be completed within a 2-month time-frame. One concern that is shared by all relates to the labeling of P rats as rats “with a family history of excessive drinking”. That is not technically accurate as there is no evidence that the parents of the P rats employed in this study consumed more alcohol than the parents of the Wistar animals.

Thus, title and this label should be changed accordingly to reflect the selected breeding strategies that were employed to generate the P rats used in this study. Also, both reviewers raised issues with the statistical approaches that are highlighted in each reviewer's individual comments. There is no drinking data provided for the animals employed in the report and this information should be provided to substantiate the claim that the alcohol intake of the 2 strains was comparable. Further, given that the 2-CAP task is relatively new to the alcohol field, more methodological detail should be provided as detailed in the individual reviews. Related to this, a more detailed description of how the head behavior of the animals was used to determine approach is critical to the interpretation of the results Reviewer 1 provided excellent suggestions for additional analyses of the recordings that might facilitate or at least complement cross-strain comparisons of firing patterns. The specific comments of each expert reviewer are provided below.

Reviewer 1:

The purpose of this manuscript is to examine firing of PFC neurons during presentation of cues associated with access to alcohol in a 2CAP task in Wistar and P rats. The authors hypothesized that mPFC neurons in P rats would display a stronger or more robust ‘intention’ signal compared with Wistar rats. Rodent locomotor behavior and mPFC neural firing patterns were analyzed during drinking and non-drinking trials. A number of variables related to firing patterns (e.g., d', mutual information, PCA, and state space) were analyzed and reported in the manuscript. In contrast to the hypothesis, the intention signal in the P rats was blunted when compared with the signal in Wistar rats. While these findings are of interest, critical details are missing that makes the manuscript difficult to review in its current form.

While the title and references throughout the manuscript to rats with a family history of excessive alcohol drinking is correct, one could interpret this to mean that the rats used in the study were born from parents that were heavy drinking rodents. Because cross-generational effects of alcohol drinking on the offspring are an emerging area of investigation, the authors should consider changing the language so it is clear that they are using P rats that have been bred through many generations from high drinking rats.

The authors provide an argument for using two strains that drink equal amounts of alcohol for these studies. The authors should report the actual drinking values of these rodents. It would be helpful to know whether high-drinking Wistar rats were used or low-drinking P rats. Were the rats matched for drinking from the IA home cage access that occurred prior to the 2CAP? The authors should also report the number of drinking vs non-drinking trials for each strain.

It is not made clear until the discussion section that the switch from ethanol availability to water access is more than a simple comparison of drinking water vs ethanol. The manuscript would be improved if the experimental design was presented in more detail and there was more of a discussion about what the switch from alcohol to water represents. The only mention of this is in the discussion section where the authors elude to a finding where P rats are slower to “extinguish 2CAP”. This should be clarified given this paradigm is not a traditional model for extinction learning. What exactly is being model when the alcohol-associated cue now predicts water availability? The authors refer to water as a reinforcer in the Discussion section. It's unclear how water serves as a reinforcer under these experimental conditions, especially since that cue predicting alcohol switches to water access.

While the authors provide a strong rationale for their mutual information analysis of the spiking data, it is difficult to interpret what the results imply given the heterogeneous nature of the cortical pyramidal neurons. As the data are presented, it is unclear what is driving the difference in mutual information between P and Wistar rats. Published studies have clustered heterogeneous recordings based on similar firing patterns and temporal distinctions related to presentation of stimuli. A complementary approach to the mutual information analysis would strengthen the manuscript. What % of neurons have similar firing patterns as those shown in Fig 3A1, A2, and A3? Does the percentage of neurons in these different categories differ between Wistar and P rats? Perhaps complementary analyses of these existing firing data will provide data to support differences between the strains.

Minor Concerns:

N values were not reported in the manuscript.

Data on the role of the mPFC in alcohol drinking from the Sommer lab should be referenced, where appropriate.

A discussion of the firing rates as reported in the Linsenbardt and Lapish, 2015 manuscript should be discussed in relation to the current results.

It is unclear how it was determined that rats were actively drinking from a presumably overhead mounted camera system.

The authors should report the methods for spike detection and thresholding.

The Timme and Lapish, 2018 manuscript does not appear to contain data showing that firing-rate distributions are highly non-normal. Please clarify or update this reference.

What subregion of the medial prefrontal cortex was targeted in this manuscript? If both prelimbic and infralimbic cortices were included, the authors should consider reporting any differences in the intention signal.

It appears that the data in this manuscript were originally published in a previous study. This should be made clear to the reader early in the manuscript, perhaps in the last paragraph of the introduction.

The analysis of the number of drinking and non-drinking trials is incorrect. This should be a repeated measures two-way ANOVA (strain X trial type (drinking/non-drinking)).

Reviewer 2:

In this study, the authors identity and quantity the information encoded by PFC neurons during cued-ethanol access, in Wistar or alcohol preferring P rats.

The work consists essentially of new and more sophisticated analysis (information theory statistics) of data collected in previous (published) studies.

With this new approach, the authors were able to provide new and important insight regarding the role of PFC computations during ethanol seeking and consumption (not a simple re-hash of old data with the same conclusions). Specifically, the authors conclude that PFC encodes the intent to drink and that this signal is blunted in alcohol preferring P rats.

Overall the paper is fine, well written, remarkably easy to read given the complexity of the statistical analysis.

However, here are some points that I hope would improve the manuscript.

• The authors mention that the behavioral and general ephys results in this task have been previously described. I think the authors should state more explicitly that this study is a more in-depth analysis of already published data. The authors should also consider explaining how this work extends the conclusion these earlier studies.

• Although the behavioral data has been analyzed in details and published elsewhere, it might a good idea to remind the reader of some main points. For instance, how much EtOH do animals consume by the end of the intermittent access protocol, or in the cued access protocol? What is the percentage or drinking vs. non-drinking trials in the cued access protocol? This could simply be mentioned in the text.

• Authors report that they were able to track the position and head direction of the rats during this task. Given the importance of the proximity to the target as a determinant of reward-seeking behavior, and cue-evoked neural responses (McGinty et al., Neuron, 2013), have the authors tried to monitor the animals' location and head direction at the onset of a trial to determine whether it has any influence on the outcome of a trial; i.e. are rats more likely to drink on a given trial if they were in close proximity and facing the cue at the start of the trial?

Generally speaking, the authors should provide more quantification of the animals' behavior during a trial; not only in terms of overall speed of movement, but also in terms of location and direction of movement. This is an important point since a main argument for the hypothesis that PFC neurons encode the “intent of drinking” is that there is no overt difference in behavior at cue presentation that separate the drinking and non-drinking trials.

• The authors report a blunting of the ‘intent to drink’ signal in the PFC of P rats, compared to Wistar rats. The disengagement of PFC might indicate the automatization of ethanol-seeking in the P rats. Have the authors tried to probe the underlying associative structures (goal-directed vs. habit based) that mediate ethanol seeking in these 2 strains, in the cued-access task (with a devaluation test for instance)?

• P15: authors wrote: “no differences in the mean number of drinking (one-way ANOVA...) or non-drinking (one-way ANOVA...) trials were observed”. it's not clear what those statistics refer to; what is being compared in each ANOVA?

• Citing Seif et al. 2013 authors wrote that “loss of information originating from mPFC is necessary for the expression of devaluation-insensitive drinking”. I'm not sure how this fit with the actual findings of Seif et al. who showed that intact mPFC inputs to NAc was required for devaluation-insensitive drinking.

## References

[B1] Ames KC, Ryu SI, Shenoy KV (2014) Neural dynamics of reaching following incorrect or absent motor preparation. Neuron 81:438–451. 10.1016/j.neuron.2013.11.003 24462104 PMC3936035

[B2] Andersen RA, Cui H (2009) Intention, action planning, and decision making in parietal-frontal circuits. Neuron 63:568–583. 10.1016/j.neuron.2009.08.028 19755101

[B3] Barr RC, Nolte LW, Pollard AE (2010) Bayesian quantitative electrophysiology and its multiple applications in bioengineering. IEEE Rev Biomed Eng 3:155–168. 10.1109/RBME.2010.2089375 22275206 PMC3935245

[B4] Bechara A (2005) Decision making, impulse control and loss of willpower to resist drugs: a neurocognitive perspective. Nat Neurosci 8:1458–1463. 10.1038/nn1584 16251988

[B5] Bell RL, Rodd ZA, Lumeng L, Murphy JM, Mcbride WJ (2006) The alcohol-preferring P rat and animal models of excessive alcohol drinking. Addict Biol 11:270–288. 10.1111/j.1369-1600.2005.00029.x 16961759

[B6] Bell RL, Rodd ZA, Engleman EA, Toalston JE, Mcbride WJ (2014) Scheduled access alcohol drinking by alcohol-preferring (P) and high-alcohol-drinking (HAD) rats: modeling adolescent and adult binge-like drinking. Alcohol 48:225–234. 10.1016/j.alcohol.2013.10.004 24290311 PMC4007416

[B7] Benjamini Y, Hochberg Y (1995) Controlling the false discovery rate: a practical and powerful approach to multiple testing. J R Stat Soc Series B Stat Methodol 57:289–300.

[B8] Boulay CB, Pieper F, Leavitt M, Martinez-Trujillo J, Sachs AJ (2016) Single-trial decoding of intended eye movement goals from lateral prefrontal cortex neural ensembles. J Neurophysiol 115:486–499. 10.1152/jn.00788.2015 26561608 PMC4760465

[B9] Brass M, Lynn MT, Demanet J, Rigoni D (2013) Imaging volition: what the brain can tell us about the will. Exp Brain Res 229:301–312. 10.1007/s00221-013-3472-x 23515626

[B10] Buschman TJ, Miller EK (2014) Goal-direction and top-down control. Philos Trans R Soc Lond B Biol Sci 369:20130471. 10.1098/rstb.2013.0471 25267814 PMC4186225

[B11] Cannady R, Mcgonigal JT, Newsom RJ, Woodward JJ, Mulholland PJ, Gass JT (2017) Prefrontal cortex K_Ca_2 channels regulate mGlu5-dependent plasticity and extinction of alcohol-seeking behavior. J Neurosci 37:4359–4369. 28320841 10.1523/JNEUROSCI.2873-16.2017 PMC5413180

[B12] Catafau AM, Etcheberrigaray A, Perez DE LOS Cobos J, Estorch M, Guardia J, Flotats A, Berna L, Mari C, Casas M, Carrio I (1999) Regional cerebral blood flow changes in chronic alcoholic patients induced by naltrexone challenge during detoxification. J Nucl Med 40:19–24. 9935051

[B13] Ciccocioppo R, Angeletti S, Weiss F (2001) Long-lasting resistance to extinction of response reinstatement induced by ethanol-related stimuli: role of genetic ethanol preference. Alcohol Clin Exp Res 25:1414–1419. 10.1111/j.1530-0277.2001.tb02141.x 11696659

[B14] Corbit LH, Nie H, Janak PH (2012) Habitual alcohol seeking: time course and the contribution of subregions of the dorsal striatum. Biol Psychiatry 72:389–395. 10.1016/j.biopsych.2012.02.024 22440617 PMC3674580

[B15] Cover TM, Thomas JA (2005) Entropy, relative entropy, and mutual information In: Elements of information theory. Hoboken, NJ: Wiley, Inc.

[B16] Cowley BR, Kaufman MT, Butler ZS, Churchland MM, Ryu SI, Shenoy KV, Yu BM (2013) DataHigh: graphical user interface for visualizing and interacting with high-dimensional neural activity. J Neural Eng 10:066012. 10.1088/1741-2560/10/6/066012 24216250 PMC3950756

[B17] Cunningham JP, Yu BM (2014) Dimensionality reduction for large-scale neural recordings. Nat Neurosci 17:1500–1509. 10.1038/nn.3776 25151264 PMC4433019

[B18] Czachowski CL, Samson HH (2002) Ethanol- and sucrose-reinforced appetitive and consummatory responding in HAD1, HAD2, and P rats. Alcohol Clin Exp Res 26:1653–1661. 10.1111/j.1530-0277.2002.tb02467.x 12436053

[B19] Dalley JW, Cardinal RN, Robbins TW (2004) Prefrontal executive and cognitive functions in rodents: neural and neurochemical substrates. Neurosci Biobehav Rev 28:771–784. 10.1016/j.neubiorev.2004.09.006 15555683

[B20] Daw ND, Niv Y, Dayan P (2005) Uncertainty-based competition between prefrontal and dorsolateral striatal systems for behavioral control. Nat Neurosci 8:1704–1711. 10.1038/nn1560 16286932

[B21] Dayas CV, Liu X, Simms JA, Weiss F (2007) Distinct patterns of neural activation associated with ethanol seeking: effects of naltrexone. Biol Psychiatry 61:979–989. 10.1016/j.biopsych.2006.07.034 17098214 PMC2831298

[B22] Dezfouli A, Balleine BW (2013) Actions, action sequences and habits: evidence that goal-directed and habitual action control are hierarchically organized. PLoS Comput Biol 9:e1003364 10.1371/journal.pcbi.1003364 24339762 PMC3854489

[B23] Doya K (1999) What are the computations of the cerebellum, the basal ganglia and the cerebral cortex?. Neural Netw 12:961–974. 12662639 10.1016/s0893-6080(99)00046-5

[B24] Fitoussi A, Le Moine C, De Deurwaerdère P, Laqui M, Rivalan M, Cador M, Dellu-Hagedorn F (2015) Prefronto-subcortical imbalance characterizes poor decision-making: neurochemical and neural functional evidences in rats. Brain Struct Funct 220:3485–3496. 10.1007/s00429-014-0868-8 25134683

[B25] Fuster JM, Bressler SL (2015) Past makes future: role of pFC in prediction. J Cogn Neurosci 27:639–654. 10.1162/jocn_a_00746 25321486

[B26] Gale SD, Perkel DJ (2010) A basal ganglia pathway drives selective auditory responses in songbird dopaminergic neurons via disinhibition. J Neurosci 30:1027–1037. 10.1523/JNEUROSCI.3585-09.2010 20089911 PMC2824341

[B27] Gatto GJ, Murphy JM, Waller MB, Mcbride WJ, Lumeng L, Li TK (1987) Chronic ethanol tolerance through free-choice drinking in the P line of alcohol-preferring rats. Pharmacol Biochem Behav 28:111–115. 10.1016/0091-3057(87)90021-9 3659102

[B28] George MS, Anton RF, Bloomer C, Teneback C, Drobes DJ, Lorberbaum JP, Nahas Z, Vincent DJ (2001) Activation of prefrontal cortex and anterior thalamus in alcoholic subjects on exposure to alcohol-specific cues. Arch Gen Psychiatry 58:345–352. 10.1001/archpsyc.58.4.345 11296095

[B29] Groblewski PA, Ryabinin AE, Cunningham CL (2012) Activation and role of the medial prefrontal cortex (mPFC) in extinction of ethanol-induced associative learning in mice. Neurobiol Learn Mem 97:37–46. 10.1016/j.nlm.2011.09.001 21951632 PMC3246036

[B30] Grüsser SM, Wrase J, Klein S, Hermann D, Smolka MN, Ruf M, Weber-Fahr W, Flor H, Mann K, Braus DF, Heinz A (2004) Cue-induced activation of the striatum and medial prefrontal cortex is associated with subsequent relapse in abstinent alcoholics. Psychopharmacology (Berl) 175:296–302. 10.1007/s00213-004-1828-4 15127179

[B31] Haynes JD, Sakai K, Rees G, Gilbert S, Frith C, Passingham RE (2007) Reading hidden intentions in the human brain. Curr Biol 17:323–328. 10.1016/j.cub.2006.11.072 17291759

[B32] Kampov-Polevoy AB, Matthews DB, Gause L, Morrow AL, Overstreet DH (2000) P rats develop physical dependence on alcohol via voluntary drinking: changes in seizure thresholds, anxiety, and patterns of alcohol drinking. Alcohol Clin Exp Res 24:278–284. 10.1111/j.1530-0277.2000.tb04608.x 10776663

[B33] Kareken DA, Bragulat V, Dzemidzic M, Cox C, Talavage T, Davidson D, O’Connor SJ (2010) Family history of alcoholism mediates the frontal response to alcoholic drink odors and alcohol in at-risk drinkers. Neuroimage 50:267–276. 10.1016/j.neuroimage.2009.11.076 20004725 PMC2819594

[B34] Keistler CR, Hammarlund E, Barker JM, Bond CW, Dileone RJ, Pittenger C, Taylor JR (2017) Regulation of alcohol extinction and cue-induced reinstatement by specific projections between medial prefrontal cortex, nucleus accumbens and basolateral amygdala. J Neurosci 37:4462–4471. 28336571 10.1523/JNEUROSCI.3383-16.2017 PMC5413184

[B35] Krawczyk DC (2002) Contributions of the prefrontal cortex to the neural basis of human decision making. Neurosci Biobehav Rev 26:631–664. 10.1016/S0149-7634(02)00021-0 12479840

[B36] Li TK, Mcbride WJ (1995) Pharmacogenetic models of alcoholism. Clin Neurosci 3:182–188. 8612063

[B37] Linsenbardt DN, Lapish CC (2015) Neural firing in the prefrontal cortex during alcohol intake in alcohol-preferring “P” versus Wistar rats. Alcohol Clin Exp Res 39:1642–1653. 10.1111/acer.12804 26250465 PMC4558392

[B38] Lumeng L, Li TK (1986) The development of metabolic tolerance in the alcohol-preferring P rats: comparison of forced and free-choice drinking of ethanol. Pharmacol Biochem Behav 25:1013–1020. 10.1016/0091-3057(86)90079-1 3786353

[B39] Mcbride WJ, Li TK (1998) Animal models of alcoholism: neurobiology of high alcohol-drinking behavior in rodents. Crit Rev Neurobiol 12:339–369. 10.1615/CritRevNeurobiol.v12.i4.40 10348615

[B40] Mcbride WJ, Rodd ZA, Bell RL, Lumeng L, Li TK (2014) The alcohol-preferring (P) and high-alcohol-drinking (HAD) rats–animal models of alcoholism. Alcohol 48:209–215. 10.1016/j.alcohol.2013.09.044 24268381 PMC4006324

[B41] Mccane AM, Czachowski CL, Lapish CC (2014) Tolcapone suppresses ethanol intake in alcohol-preferring rats performing a novel cued access protocol. Alcohol Clin Exp Res 38:2468–2478. 10.1111/acer.12515 25257296 PMC4260468

[B42] Momennejad I, Haynes JD (2013) Encoding of prospective tasks in the human prefrontal cortex under varying task loads. J Neurosci 33:17342–17349. 10.1523/JNEUROSCI.0492-13.2013 24174667 PMC6618369

[B43] Myrick H, Anton RF, Li X, Henderson S, Drobes D, Voronin K, George MS (2004) Differential brain activity in alcoholics and social drinkers to alcohol cues: relationship to craving. Neuropsychopharmacology 29:393–402. 10.1038/sj.npp.1300295 14679386

[B44] National Research Council (2003) Guidelines for the care and use of mammals in neuroscience and behavioral research The National Academies Press: Washington, DC. 20669478

[B45] Panzeri S, Senatore R, Montemurro MA, Petersen RS (2007) Correcting for the sampling bias problem in spike train information measures. J Neurophysiol 98:1064–1072. 10.1152/jn.00559.2007 17615128

[B46] Pfarr S, Meinhardt MW, Klee ML, Hansson AC, Vengeliene V, Schonig K, Bartsch D, Hope BT, Spanagel R, Sommer WH (2015) Losing control: excessive alcohol seeking after selective inactivation of cue-responsive neurons in the infralimbic cortex. J Neurosci 35:10750–10761. 10.1523/JNEUROSCI.0684-15.2015 26224858 PMC6605108

[B47] Rangel A, Camerer C, Montague PR (2008) A framework for studying the neurobiology of value-based decision making. Nat Rev Neurosci 9:545–556. 10.1038/nrn2357 18545266 PMC4332708

[B48] Redish AD, Jensen S, Johnson A (2008) A unified framework for addiction: vulnerabilities in the decision process. Behav Brain Sci 31:415–437, discussion 437–487 10.1017/S0140525X0800472X 18662461 PMC3774323

[B49] Ridderinkhof KR, VAN DEN Wildenberg WP, Segalowitz SJ, Carter CS (2004) Neurocognitive mechanisms of cognitive control: the role of prefrontal cortex in action selection, response inhibition, performance monitoring, and reward-based learning. Brain Cogn 56:129–140. 10.1016/j.bandc.2004.09.016 15518930

[B50] Roxin A, Brunel N, Hansel D, Mongillo G, VAN Vreeswijk C (2011) On the distribution of firing rates in networks of cortical neurons. J Neurosci 31:16217–16226. 10.1523/JNEUROSCI.1677-11.2011 22072673 PMC6633220

[B51] Sakagami M, Niki H (1994) Encoding of behavioral significance of visual stimuli by primate prefrontal neurons: relation to relevant task conditions. Exp Brain Res 97:423–436. 10.1007/BF00241536 8187854

[B52] Sakagami M, Tsutsui K (1999) The hierarchical organization of decision making in the primate prefrontal cortex. Neurosci Res 34:79–89. 10.1016/S0168-0102(99)00038-3 10498334

[B53] Schacht JP, Anton RF, Myrick H (2013) Functional neuroimaging studies of alcohol cue reactivity: a quantitative meta-analysis and systematic review. Addict Biol 18:121–133. 10.1111/j.1369-1600.2012.00464.x 22574861 PMC3419322

[B54] Simms JA, Steensland P, Medina B, Abernathy KE, Chandler LJ, Wise R, Bartlett SE (2008) Intermittent access to 20% ethanol induces high ethanol consumption in Long-Evans and Wistar rats. Alcohol Clin Exp Res 32:1816–1823. 10.1111/j.1530-0277.2008.00753.x 18671810 PMC3151464

[B55] Stewart RB, Mcbride WJ, Lumeng L, Li TK, Murphy JM (1991) Chronic alcohol consumption in alcohol-preferring P rats attenuates subsequent conditioned taste aversion produced by ethanol injections. Psychopharmacology (Berl) 105:530–534. 10.1007/BF02244375 1771221

[B56] Tanji J, Hoshi E (2001) Behavioral planning in the prefrontal cortex. Curr Opin Neurobiol 11:164–170. 11301235 10.1016/s0959-4388(00)00192-6

[B57] Tapert SF, Cheung EH, Brown GG, Frank LR, Paulus MP, Schweinsburg AD, Meloy MJ, Brown SA (2003) Neural response to alcohol stimuli in adolescents with alcohol use disorder. Arch Gen Psychiatry 60:727–735. 10.1001/archpsyc.60.7.727 12860777

[B58] Timme NM, Lapish C (2018) A tutorial for information theory in neuroscience. eNeuro 5:ENEURO.0052-18.2018. 10.1523/ENEURO.0052-18.2018 30211307 PMC6131830

[B59] Timme NM, Marshall NJ, Bennett N, Ripp M, Lautzenhiser E, Beggs JM (2016) Criticality maximizes complexity in neural tissue. Front Physiol 7:425. 27729870 10.3389/fphys.2016.00425 PMC5037237

[B60] Treves A, Panzeri S (1995) The upward bias in measures of information derived from limited data samples. Neural Comput 7:399–407. 10.1162/neco.1995.7.2.399

[B61] Verdejo-Garcia A, Chong TT, Stout JC, Yücel M, London ED (2018) Stages of dysfunctional decision-making in addiction. Pharmacol Biochem Behav 164:99–105. 28216068 10.1016/j.pbb.2017.02.003

[B62] Waller MB, Mcbride WJ, Lumeng L, Li TK (1982) Induction of dependence on ethanol by free-choice drinking in alcohol-preferring rats. Pharmacol Biochem Behav 16:501–507. 10.1016/0091-3057(82)90459-2 7200611

